# Prevalence of burnout among intensivists in mainland China: a nationwide cross-sectional survey

**DOI:** 10.1186/s13054-020-03439-8

**Published:** 2021-01-05

**Authors:** Jing Wang, Bo Hu, Zhiyong Peng, Huimin Song, Shuhan Cai, Xin Rao, Lu Li, Jianguo Li

**Affiliations:** 1 Department of Critical Care Medicine, Wuhan University Zhongnan Hospital, Wuhan, China; 2 Clinical Research Center of Hubei Critical Care Medicine, Wuhan, China

**Keywords:** Burnout rate, Intensivists, Mainland china, Self-administered questionnaire survey

## Abstract

**Background:**

Burnout has gained increasing attention worldwide; however, there is a lack of relevant research in China. This study investigated the prevalence and factors associated with burnout in physicians of the intensive care unit (ICU) in mainland China.

**Methods:**

This cross-sectional multicenter study included critical care physicians from all provinces in mainland China (except Tibet). A self-administered survey questionnaire was conducted. It included three parts: demographic information, lifestyle and work information, and the Maslach Burnout Inventory. The levels of burnout were calculated. The factors independently associated with burnout were analyzed by logistic regression.

**Results:**

Finally, 1813 intensivists participated in the survey. The participation rate was 90.7%. The prevalence of burnout and severe burnout was 82.1% (1489/1813) and 38.8% (704/1813), respectively. According to the logistic regression analysis, “difficulty in making treatment decisions” was independently associated with burnout [OR = 1.365, CI (1.060, 1.757)]. “Higher number of children” [OR = 0.714, CI (0.519, 0.981)] and higher “income satisfaction” [OR = 0.771, CI (0.619, 0.959)] were independent protective factors against severe burnout.

**Conclusions:**

The burnout rate in ICU physicians in China is high. Difficult treatment decisions, the number of children, and income satisfaction are independently associated with burnout rates among ICU physicians in China.

*Trial registration*: Burnout syndrome of the Chinese personnel working in intensive care units: a survey in China, ChiCTR-EOC-17013044, registered October 19, 2017. http://www.chictr.org.cn/showproj.aspx?proj=22329.

## Background

Burnout is defined as a state of physical and emotional exhaustion caused by excessive and sustained levels of work-related stress [1]. It usually manifests as emotional exhaustion, depersonalization, and a reduced sense of personal accomplishments [2]. The detrimental effects of burnout are important not only for the mental health of physicians but also for the safety and quality of patient care. Moreover, this adverse influence of burnout is associated with a high occurrence of healthcare-associated infections [3]. The risk factors associated with burnout are female sex, moral distress, conflicts with colleagues, long working hours, highly demanding work, and decision on treatment withdrawal, among others [4–9]. In contrast, practicing sports, psychological rumination, resilience training programs, and small-group physician curriculums are useful strategies to decrease the incidence of burnout [6, 8, 10, 11].

Medical providers, especially those working in intensive care units (ICUs), are at high risk of developing burnout due to high levels of work stress, intense work intensity, and exhaustion. According to a previous survey, the burnout rate of critical care professionals accounts for 53% of all medical specialists [12]. The increasing prevalence of burnout and its detrimental effects on caregivers, patients, and the whole medical community are under intensive focus. In 2016, a call for action against the burnout syndrome in critical care health care professionals was issued by an official collaborative society for critical care [13, 14].

Many studies on the epidemiology, risk factors, and treatment strategies for burnout have been conducted in Brazil, Portugal, Poland, the USA, and other countries [3–7, 10, 15–18], but studies on the epidemiology of burnout of ICU physicians in China are extremely limited [19–21]. The respondents in the three published studies were from only one or two provinces of China, limiting the generalizability of the results. In addition, ICU physicians were not included in the investigated populations. Thus, it is imperative to conduct a nationwide survey looking at the prevalence of burnout among Chinese intensivists and to explore the associated and preventive factors. We conducted such a survey under the support of Chinese Society of Critical Care Medicine.

## Materials and methods

### Study design

The present cross-sectional multicenter study covered all provinces of mainland China, except Tibet. A self-completed questionnaire was distributed in all provinces by the personnel chosen by the Critical Care Medicine Branch of the Chinese Medical Association. The survey was conducted between December 13, 2017, and October 19, 2019.

This study protocol was approved by the Medical Ethics Committee of Wuhan University Zhongnan Hospital (2017015). Informed consent was obtained from all individual participants. The clinical trial registration number is ChiCTR-EOC-17013044 (http://www.chictr.org.cn/showproj.aspx?proj=22329). All the respondents were willing to participate in the study, and the questionnaires were anonymous.

### Incision and exclusion criteria

The inclusion criteria of the hospitals were (1) general hospital, (2) hospital grade of secondary or above, and (3) the hospital has an ICU. The exclusion criterion was that a specialized hospital could not be considered. The inclusion criteria of the respondents were (1) at least 1 year of work experience at the surveyed hospital, (2) currently involved in clinical medical work, and (3) willing to participate in the survey. The exclusion criteria of the respondents were (1) involved in non-clinical medical work at the same time or (2) worked at a specialized ICU.

### Questionnaire

The questionnaire was designed by an expert team consisting of intensivists, epidemiologists, statisticians, a psychologist, and hospital managers. These experts were all professors who had been working for > 10 years at Wuhan University. The questionnaire had three parts: (1) demographic information: age, sex, marital status, number of children, family history of mental disease, educational background, academic title, and length of service; (2) lifestyle, work status, and other factors that might be associated with burnout: uncomfortable symptoms, chronic diseases, source of pressures, ways to relieve stress, weekly working hours, night shift (the frequency of on duty at night), daily commuting time (average time spent each day commuting to hospital), the holidays (having or not official annual vacations), acceptability of working on holidays, compensation for working on holidays, satisfaction with income, conflicts with colleagues, difficulties in treatment decisions (physicians in ICU often have to face the choice of giving up or continuing with futile therapy when considering the medical costs and ethics), worries related to safety (injury to physicians caused by a poor physician–patient relationship often make physicians unsafe), medical complaint affairs (complaints from patients and their families about dissatisfaction with hospitals, departments, and medical staff), reasons for choosing the ICU, satisfaction with working in the ICU, and consideration of turnover and reasons; 3) the Maslach Burnout Inventory (MBI), which is regarded as the optimal burnout assessment tool [22]. The survey is presented as Additional file 1: Table 1.

The MBI that was used in this study to evaluate the prevalence of burnout is in compliance with the international standards [8]. It consists of 22 items across three categories: emotional exhaustion (EE), depersonalization (DP), and decreased personal accomplishment (PA). The scores for EE were ≤ 18 (low degree), 19–26 (moderate degree), and ≥ 27 (severe degree). The scores for DP were ≤ 5 (low degree), 6–9 (moderate degree), and ≥ 10 (severe degree). The scores for PA were ≥ 40 (low degree), 34–39 (moderate degree), and ≤ 33 (severe degree).

### Outcome

The outcome of this study was the positive burnout of respondents, according to the MBI. The definition of positive burnout in this study was determined by a high score of EE or DP or a low score of PA. In this study, severe burnout was defined as a high score of EE, along with a high score of DP or a low score of PA [23–25]. The total scores of EE, DP, and PA in every questionnaire were calculated by two data analysts. The results were checked and corrected in the case of discrepancies between data analysts.

### Procedure

In China, the Critical Care Medicine Departments are managed by the Critical Care Medicine Branch of the Chinese Medical Association, which monitor those in general and not specialized ICUs. The academic instructors of the Critical Care Medicine departments of all provinces and cities in China were from the Critical Care Medicine Branch of the Chinese Medical Association, and the Standing Committee of this branch was from all provinces across the country, representing the highest academic leader in the province. The Standing Committee members of each province, municipality, or autonomous region published the requirements of this survey in the WeChat group of the hospitals with ICUs in their respective provinces according to the inclusion criteria and exclusion criteria of the hospital. The Standing Committee members issued the questionnaires to the hospitals that met the standards and were willing to participate in the investigation. The hospital organized the survey respondents to fill out the questionnaire. The electronic questionnaires were created in the platform of “The Questionnaire Star” (https://www.wjx.cn), and a unique two-dimensional code was provided. The study personnel in each province distributed the questionnaires to the ICU physicians of their province. The release and submission of questionnaires could be checked on the platform.

### Statistical analysis

The categorical variables are expressed as frequencies (n) and percentages (%). A normality test was used for continuous variables. Means and standard deviation are used to present the variables with a normal distribution; median and percentiles are used when the variables are not normally distributed. The chi-square test was used to examine the associations among the categorical variables. The *P* value for statistically significant differences was set at < 0.05. Fisher’s exact test was used for an expected frequency in any cell of a contingency table of < 5. Regression analysis was performed to explore the factors associated with burnout. All analyses were performed using SPSS v.24.0 (IBM Co., Armonk, NY, USA).

## Results

A total of 2000 questionnaires were distributed in the 30 provinces and autonomous regions of mainland China (except Tibet). Additional file 2: Table 2 lists the hospitals and respondents participating in the study. Of these, 1813 were completed and returned. The response rate was 90.7%. The social and demographic characteristics of the respondents are presented in Table 1.

**Table 1:** The social and demographic characteristics of the respondents

Information and characteristics	*N* = 1813	%
Grade of hospital		
Tertiary	1195	65.9
Secondary	618	34.1
Teaching hospital		
Yes	699	38.5
Beds in hospital		
< 1000	973	53.7
1000–2000	601	33.2
> 2000	239	13.2
Beds in ICU		
< 10	590	32.5
10–20	796	43.9
> 20	427	23.6
Sex		
Male	1140	62.9
Female	673	37.1
Age		
≤ 30	216	11.9
31–39	818	45.1
≥ 40	779	43.0
Marital status		
Married	1670	92.1
Single or others	143	7.9
Children		
None	250	13.8
One	1091	60.2
≥ 2 children	472	26.0
Family history of mental disease		
Yes	44	2.4
Educational background		
Undergraduate	1221	67.4
Postgraduate	592	32.7
Academic title		
Resident	352	19.4
Attending physicians	696	38.4
Directors	765	42.2
Length of service in ICU		
1–5 years	580	32.0
5–10 years	672	37.1
> 10 years	561	30.9

Variables are expressed as numbers (percentages)

### Prevalence of burnout

**Fig. 1:**
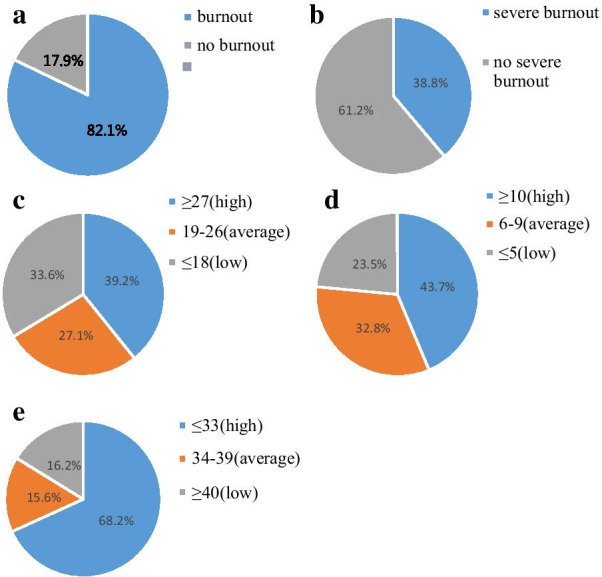
The rate of burnout and severe burnout in China. **a** The rate of burnout in China; **b** the rate of severe burnout in China; **c** the rate of intensivists in each level of emotional exhaustion; **d** the rate of intensivists in each level of depersonalization; **f** the rate of intensivists in each level of personal accomplishment. Variables are expressed as numbers (percentages)

In this survey, intensivists in China showed a high prevalence of burnout, at 82.1% (1489/1813), and the prevalence of severe burnout was 38.8% (704/1813) (Fig. 1a, b). The rate of intensivists in the three dimensions of burnout is shown in Fig. 1c–e. The mean scores of the three dimensions of burnout were 24.14 ± 10.90 in EE, 9.69 ± 5.70 in depersonalization, and 28.55 ± 9.82 in PA. It suggests that the EE and depersonalization levels were both moderate, but that the PA level was high.

### Factors associated with burnout

#### Relationship between personal information and respondent burnout

As shown in Table 2, age is associated with burnout (*P* < 0.001) and severe burnout (*P* < 0.001). The number of children of the respondents was associated with burnout (*P* = 0.004) and severe burnout (*P* < 0.001). The respondents with a family history of mental diseases had a higher prevalence of burnout (*P* = 0.02) and severe burnout (*P* = 0.03). The length of service in the ICU was also associated with burnout (*P* = 0.006) and severe burnout (*P* < 0.001).

**Table 2:** Personal characteristics associated with burnout and severe burnout

Personal characteristics	Total, *n* (%)	Burnout, *n* (%)	Severe burnout, *n* (%)
Sex			
Male	1140 (62.9%)	922 (80.9%)	403 (35.4%)
Female	673 (37.1%)	567 (84.3%)	301 (44.7%)
		*P* = 0.07	*P* < 0.001
Age			
≤ 30 years	216 (11.9%)	192 (88.9%)	104 (48.2%)
31–39 years	818 (45.1%)	699 (85.5%)	353 (43.2%)
≥ 40 years	779 (43.0%)	598 (76.8%)	247 (31.7%)
		*P* < 0.001	*P* < 0.001
Marital status			
Married	1670 (92.1%)	1369 (81.9%)	634 (38.0%)
Single or others	143 (7.9%)	120 (83.9%)	70 (49.0%)
		*P* = 0.561	*P* = 0.010
Children			
None	250 (13.8%)	224 (89.6%)	133 (53.2%)
One	1091 (60.2%)	884 (81.0%)	421 (38.6%)
≥ 2 children	472 (26.0%)	381 (80.7%)	150 (31.8%)
		*P* = 0.004	*P* < 0.001
Family history of mental disease			
Yes	44 (2.4%)	42 (95.5%)	24 (54.6%)
		*P* = 0.02	*P* = 0.030
Educational background			
Undergraduate	1221 (67.4%)	1006 (82.4%)	456 (37.4%)
Postgraduate	592 (32.7%)	483 (81.6%)	248 (41.9%)
		*P* = 0.675	*P* = 0.063
Academic title			
Resident	352 (19.4%)	300 (85.2%)	165 (46.9%)
Attending physicians	696 (38.4%)	597 (85.8%)	293 (42.1%)
Directors	765 (42.2%)	592 (77.4%)	246 (32.2%)
		*P* < 0.001	*P* < 0.001
Length of service			
1–5 years	580 (32.0%)	496 (85.5%)	258 (44.5%)
6–10 years	672 (37.1%)	554 (82.4%)	260 (38.7%)
> 10 years	561 (30.9%)	439 (62.2%)	186 (33.2%)
		*P* = 0.006	*P* < 0.001

Variables are expressed as numbers (percentages). *P* < 0.05 is regarded as a significant difference

In addition, female respondents exhibited a higher prevalence of severe burnout than males (*P* < 0.001). Married respondents had a lower prevalence of severe burnout than singles (*P* = 0.010). Nevertheless, no difference was observed in the prevalence of burnout between sexes and marital statuses (*P* = 0.07; *P* = 0.561). In addition, the educational background did not show any association with the prevalence of burnout and severe burnout (*P* = 0.675; *P* = 0.063) (Table 2).

#### Relationship between professional characteristics and burnout

Intensive work is a prominent feature of ICUs, but no difference in the prevalence of burnout and severe burnout was observed among physicians who worked for ≤ 40, 41–60, and > 60 h (*P* = 0.300; *P* = 0.064). Physicians who had night shifts had a different prevalence of burnout (*P* < 0.001) and severe burnout (*P* < 0.001). The respondents with shorter commuting time (≤ 0.5 h) had a lower prevalence of burnout (*P* = 0.003) and severe burnout (*P* = 0.001) than those with > 0.5 h of commuting (Table 3).

**Table 3:** Workplace characteristics and work attitude of respondents associated with burnout

Work experiences	*n* (%)	Burnout		Severe burnout	
		%	*P*/*X*^2^	%	*P*/*X*^2^
Weekly working hours					
≤ 40 h	65 (3.6%)	53 (81.5%)	*P* = 0.300	23 (35.4%)	*P* = 0.064
41–60 h	932 (51.4%)	778 (83.5%)		340 (36.5%)	
> 60 h	816 (45.0%)	658 (80.6%)	*X*^2^ = 2.405	341 (41.8%)	*X*^2^ = 5.499
Night shift^a^					
One every ≤ 4 working days	927 (51.1%)	768 (82.9%)	*P* < 0.001	362 (39.1%)	*P* < 0.001
One every 5–7 d working days	669 (36.9%)	564 (84.3%)	*X*^2^ = 16.622	294 (44.0%)	*X*^2^ = 32.902
One every > 7 d working days	217 (12.0%)	157 (72.4%)		48 (22.1%)	
Daily commuting time^b^					
≤ 0.5 h	1200 (66.2%)	963 (80.3%)	*P* < 0.001	433 (36.7%)	*P* < 0.001
> 0.5 h	613 (33.8%)	526 (85.8%)	*X*^2^ = 8.538	271 (29.9%)	*X*^2^ = 11.278
Holidays^c^					
No holidays	1218 (67.2%)	1010 (82.9%)	*P* = 0.207	482 (39.6%)	*P* = 0.353
Having holidays	595 (32.8%)	479 (80.5%)	*X*^2^ = 1.593	222 (37.3%)	*X*^2^ = 0.861
Attitude to work on holidays					
Acceptance	1059 (58.4%)	818 (77.2%)	*P* < 0.001	326 (30.8%)	*P* < 0.001
Not acceptance	433 (23.9%)	401 (92.6%)	*X*^2^ = 50.493	252 (58.2%)	*X*^2^ = 97.277
Indifferent	321 (17.7%)	270 (84.1%)		126 (39.3%)	
Compensation for working on holidays					
No compensation	1275 (70.3%)	1064 (83.5%)	*P* = 0.024	532 (41.7%)	*P* < 0.001
Having compensation	538 (29.8%)	425 (79.0%)	*X*^2^ = 5.115	173 (32.0%)	*X*^2^ = 15.16
Satisfaction with income					
Yes	481 (26.5%)	359 (74.6%)	*P* < 0.001	147 (30.6%)	*P* < 0.001
No	1332 (73.5%)	1130 (84.8%)	*X*^2^ = 25.043	557 (41.8%)	*X*^2^ = 18.848
Conflict with colleagues					
Rare	1684 (92.9%)	1368 (81.2%)	*P* < 0.001	632 (37.5%)	*P* < 0.001
Often	129 (7.1%)	121 (93.8%)	*X*^2^ = 12.885	72 (55.8%)	*X*^2^ = 16.157
Difficulties in treatment decisions					
Rare	1095 (60.4%)	855 (78.1%)	*P* < 0.001	356 (32.5%)	*P* < 0.001
Often	718 (39.6%)	634 (88.3%)	*X*^2^ = 30.852	348 (48.5%)	*X*^2^ = 46.485
Worries related to safety					
Rare	1002 (55.3%)	766 (76.5%)	*P* < 0.001	296 (29.5%)	*P* < 0.001
Often	811 (44.7%)	723 (89.2%)	*X*^2^ = 49.272	408 (50.3%)	*X*^2^ = 81.385
Medical complain affairs^d^					
No	1776 (98.0%)	1454 (80.2%)	*P* = 0.046	679 (38.2%)	*P* < 0.001
Yes	372 (2.0%)	359 (94.6%)	*X*^2^ = 3.999	25 (67.5%)	*X*^2^ = 13.132
Reasons for choice of ICU					
Affinity	519 (28.6%)	377 (72.6%)	*P* < 0.001	155 (29.9%)	*P* < 0.001
Distribution	1083 (59.7%)	926 (85.5%)	*X*^2^ = 45.457	449 (41.5%)	*X*^2^ = 27.226
Others	211 (11.6%)	186 (88.2%)		100 (47.4%)	
Satisfaction with ICU					
More love	821 (44.3%)	583 (52.0%)	*P* < 0.001	224 (27.3%)	*P* < 0.001
Less love	258 (29.8%)	240 (84.6%)	*X*^2^ = 127.05	168 (65.1%)	*X*^2^ = 125.31
The same as before	734 (25.9%)	666 (78.8%)		312 (42.5%)	
Turnover consideration					
No	996 (54.9%)	767 (77.0%)	*P* < 0.001	266 (26.7%)	*P* < 0.001
Yes	693 (38.2%)	616 (88.9%)	*X*^2^ = 40.323	377 (54.4%)	*X*^2^ = 137.97
Not sure	124 (6.8%)	106 (85.5%)		61 (49.2%)	
Reason for stay					
A love of this work	709 (80.3%)	495 (83.2%)	*P* < 0.001	181 (39.4%)	*P* < 0.001
No other choice	906 (80.3%)	828 (81.1%)	*X*^2^ = 126.57	424 (36.4%)	*X*^2^ = 87.434
Other reasons	198 (56.7%)	166 (82.5%)		99 (41.1%)	

^a^The frequency of on duty at night every < 4, 5–7, or > 7 days

^b^The average time spent each day commuting to the hospital

^c^The official annual vacations

^d^Complaints from patients and their families about dissatisfaction with hospitals, departments, and medical staff

In this survey, 1218 physicians (67.2%) had no holidays, but this did not affect the prevalence of burnout and severe burnout (*P* = 0.207, *P* = 0.353). The attitude towards work on holidays was associated with burnout (*P* < 0.001) and severe burnout (*P* < 0.001). Only 539 (29.7%) intensivists were compensated for working on holidays, and they had a significantly lower prevalence of burnout and severe burnout than those who received no compensation (*P* = 0.019; *P* < 0.001) (Table 3).

In this survey, 1132 (73.5%) physicians were not satisfied with their income. Their prevalence of burnout (*P* < 0.001) and severe burnout (*P* < 0.001) was significantly higher than that of those who were satisfied with their income. During daily work, the physicians who often had conflicts with colleagues had a higher prevalence of burnout (*P* < 0.001) and severe burnout (*P* < 0.001). Those who often had confusion with treatment had a higher prevalence of burnout (*P* < 0.001) and severe burnout (*P* < 0.001). Respondents who often worried about their personal safety displayed a higher prevalence of burnout (*P* < 0.001) and severe burnout (*P* < 0.001). Respondents who had medical complaint affairs were characterized by high rates of burnout (*P* = 0.046) and severe burnout (*P* < 0.001) compared to those who rarely experienced these problems (Table 3).

More than 50% (1083/59.7%) of the respondents chose the department of ICU because of their postgraduate distribution. Only 519 (28.6%) chose it for their affinities. The different reasons for choosing of ICU were associated with the prevalence of burnout (*P* < 0.001) and severe burnout (*P* < 0.001). Interestingly, 821 (44.3%) of the intensivists loved their work at the ICU even more than before, which was associated with burnout (*P* < 0.001) and severe burnout (*P* < 0.001). The survey showed that 996 (54.9%) intensivists had no consideration of turnover. Different attitudes in turnover were associated with burnout (*P* < 0.001) and severe burnout (*P* < 0.001). Among the intensivists not considering turnover, 709 (80.31%) did so due to their love for the work, and this was associated with (*P* < 0.001) and severe burnout (*P* < 0.001) (Table 3).

#### Independent factors associated with burnout and severe burnout

**Fig. 2:**
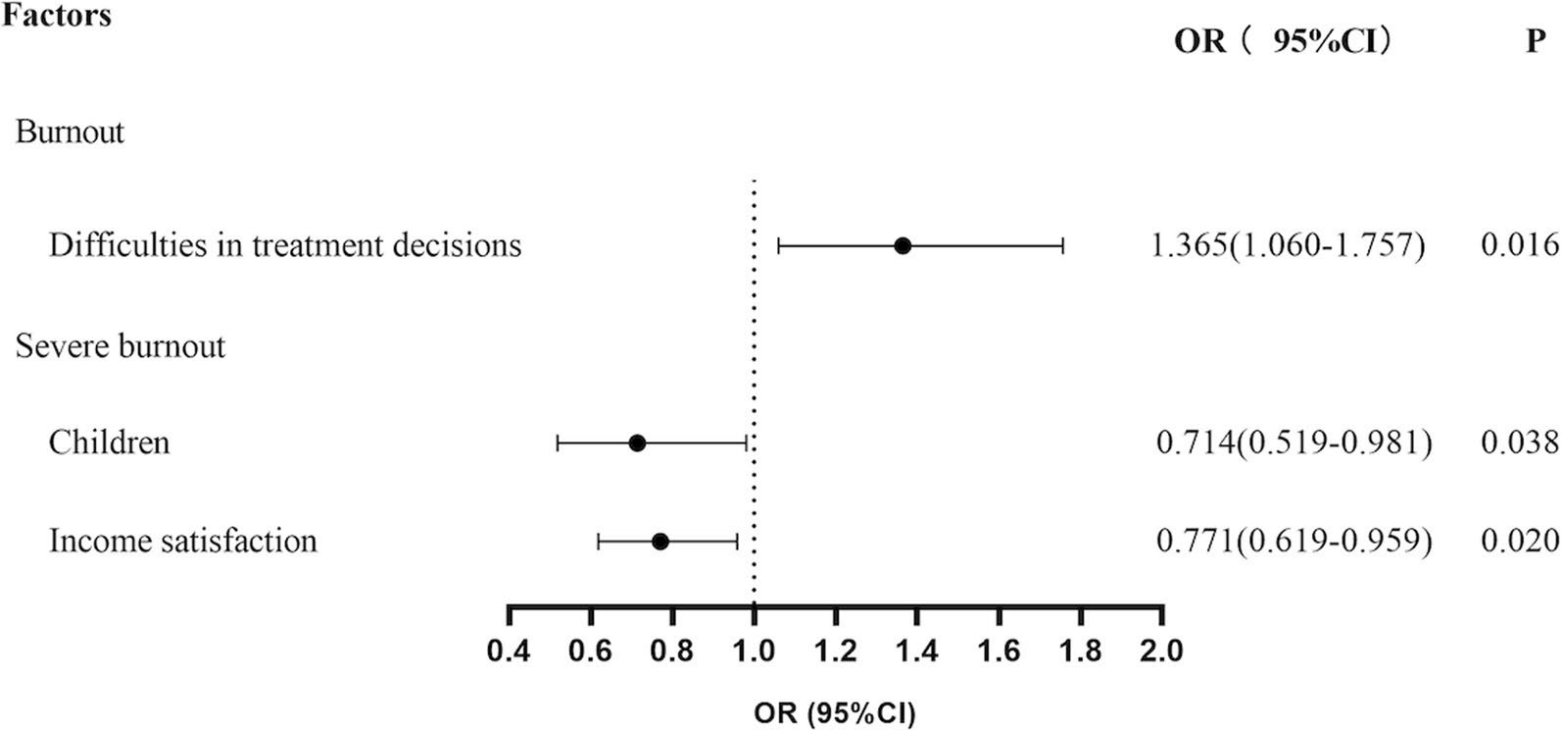
Analysis of risk factors for burnout and severe burnout

Regression analysis was used to explore the independent factors for burnout and severe burnout. The following factors were examined: sex, age, marital status, children, family history of mental disease, academic title, length of service, night shifts, commuting time, attitude to work on holidays, compensation for working on holidays, satisfaction with income, conflict with colleagues, difficulties in treatment decisions, worries related to safety, medical complain affairs, reasons for the choice of ICU, satisfaction with ICU, turnover consideration, the reason for the stay. The results revealed that difficulties in treatment decisions were associated with burnout (*P* = 0.016), while having children (*P* = 0.038) and satisfaction with income (*P* = 0.020) were associated with severe burnout in the univariable regression analysis. In the multiple regression analysis, difficulties in treatment decisions [OR = 1.365, CI (1.060, 1.757)], higher number of children [OR = 0.714, CI (0.519, 0.981)], and income satisfaction [OR = 0.771,CI (0.619, 0.959)] were independently associated with severe burnout (Fig. 2).

#### Health status and pressure sources of respondents

In the present survey, the top three uncomfortable symptoms of the respondents included fatigue, insomnia, and dizziness/headache. The prevalence of burnout and severe burnout in respondents who had fatigue, insomnia, and dizziness/headache is presented in Table 4. The most common chronic diseases of the respondents included chronic sleep disorder (468/25.8%), hepatic steatosis (450/24.8%), and lumbar disk herniation (386/21.3%). On the other hand, the top three sources of pressure included research and promotion requirements (942/52.0%), clinical practice (523/28.9%), and family burden (164/9.1%). Furthermore, the most popular ways to relieve stress included sleep (1031/56.9%), sports (887/48.9%), and leisure and recreation activities (780/43.0%) (Table 4).

**Table 4:** Health and work satisfaction and the rate of burnout and severe burnout

Items	*N*	Burnout, *n* (%)	Severe burnout, *n* (%)
Uncomfortable symptoms			
Fatigue	1589 (87.6%)	1347 (84.8%)	654 (41.2%)
Insomnia	989 (54.6%)	855 (86.5%)	457 (46.2%)
Dizziness or headache	931 (51.4%)	811 (87.1%)	433 (46.5%)
Chronic diseases			
No	607 (33.5%)	472 (77.8%)	209 (34.4%)
Chronic sleep disorder	468 (25.8%)	415 (88.7%)	241 (51.5%)
Hepatic adipose infiltration	450 (24.8%)	374 (83.1%)	192 (42.7%)
Lumbar disk protrusion	386 (21.3%)	326 (84.5%)	160 (41.5%)
Source of pressures			
Research and promotion requirements	942 (52.0%)	761 (80.8%)	332 (35.2%)
Clinical practice	523 (28.9%)	447 (85.5%)	242 (46.3%)
Family burden	164 (9.1%)	138 (84.2%)	59 (36.0%)
Ways to relieve stress			
Sleep	1031 (56.9%)	874 (84.8%)	451 (43.7%)
Sports	887 (48.9%)	683 (77.0%)	288 (32.5%)
Leisure and recreation	780 (43.0%)	649 (83.2%)	304 (39.0%)
Most dissatisfaction			
Income	1456 (80.3%)	1212 (83.2%)	574 (39.4%)
Lack of medical resources	1455 (80.3%)	1180 (81.1%)	530 (36.4%)
Working environment	1028 (56.7%)	848 (82.5%)	422 (41.1%)
Most unwilling to do			
Non-academic conference	1352 (77.2%)	1105 (81.7%)	519 (38.4%)
Taking patient out for examination	1135 (64.0%)	932 (82.1%)	431 (38.0%)
Medical record writing	932 (46.1%)	775 (83.2%)	346 (37.1%)
Factors affecting income			
Clinical workload	1252 (69.1%)	1030 (82.3%)	478 (38.2%)
Working qualifications	1001 (55.2%)	826 (82.5%)	375 (37.5%)
Drug ratio penalty	795 (43.9%)	657 (82.6%)	326 (41.0%)

Variables are expressed as numbers (percentages)

The highest dissatisfaction of the respondents was associated with their income (1456/80.3%), lack of medical resources (1455/80.3%), and their working environment (1028/56.7%). The top three influences associated with income included clinical workload (1252/69.1%), working qualifications (1001/55.2%), and drug ratio penalty (795/43.9%). In terms of daily work duties, the respondents were mostly unwilling to attend non-academic conferences (1352/77.2%), taking the patient out for an examination (1135/64.0%), and medical record writing (932/46.1%) (Table 4).

## Discussion

Intensivists frequently face the problem of burnout due to the pressure coming from patients, family, and work. Therefore, we focused on the intensivists’ burnout in China, aiming to call for attention and to enhance their work enthusiasm. This is the first nationwide burnout study about ICU physicians in China. Through the survey, we found that the prevalence of burnout among ICU physicians in China was > 80% and that the prevalence of severe burnout was almost 40%. Among the factors associated with burnout, difficulty in treatment decisions was associated with burnout, while the number of children and income satisfaction was associated with severe burnout. With wide coverage and strong representation, this study provides first-hand epidemiological information that serves as a foundation for further studies on burnout in China.

The results showed that the prevalence of burnout rate in intensivists in China was very high and even higher than those reported in previous studies in Western countries [3–7, 10, 15–18]. The prevalence observed here is also higher than that in previous Chinese studies [19–21], but those studies were limited to specific regions or cities. Nevertheless, a recent meta-analysis reveals that 40% emergency medicine physicians have high levels of EE and DP [26]. The differences might be due to fundamental differences in the medical systems between China and the West. Meanwhile, many physicians in China do not know about burnout or even never heard about it before. As a result, the issue of burnout in Chinese intensivists is ignored and requires wide attention.

Some factors are associated with the prevalence of burnout. Previous studies have revealed that work environment, occupational stress, work conditions, workload, quality of life, health, and work activity are considered major burnout-related factors [27–32]. To explore the possible factors associated with burnout in China, the aspects described above were taken into consideration and included in the questionnaire used in this study. In this survey, many individual and job characteristics probably associated with burnout and severe burnout were included. Nevertheless, these factors might influence each other. After univariable and multiple regression analysis, the results showed that a higher number of children is a protective factor against severe burnout in ICU physicians in China. Another study in Hubei Province showed that work environment satisfaction, job rewards satisfaction [20], organization management satisfaction, and emotional exhaustion are associated with burnout, but those dimensions were not assessed in this study.

In addition to the above factors, we inferred that the high prevalence of burnout in China might be due to some Chinese-specific factors such as work attitude on holidays, reasons for choosing ICU, and job satisfaction. Our survey revealed that difficulty in treatment decisions and income satisfaction are associated with burnout and severe burnout. This would remind physicians that when facing treatment decisions, they can call for help from medical affairs or other coworkers to avoid burnout. Income satisfaction should also be taken seriously to decrease the rate of intensivists’ burnout.

In this study, we used the MBI to evaluate the prevalence of burnout. Indeed, some other measurements, like the ProQoL scale, can also be used for burnout. However, unlike the MBI scale, the ProQoL scale includes compassion and satisfaction, burnout, and secondary traumatic stress, with a total of 30 items [33]. Compassion and satisfaction refer to the happiness you feel when you complete a task. Secondary traumatic stress refers to secondary exposure to extreme or traumatic stress events. Therefore, the evaluation content of the ProQoL scale is broader than burnout, and the evaluation of burnout is only a small part of the ProQoL scale. There are only ten items related to burnout in the scale. Since the focus of this survey was burnout, and since the MBI scale is recognized as the most classic scale for evaluating burnout, the MBI scale was selected.

The present study has some limitations. First, a certain degree of arbitrariness existed in the questionnaire respondents, although they were from all regions and provinces of China (except Tibet). This bias might be due to the enrollment of a large sample size. Second, similar to all cross-sectional design studies, the results cannot provide any cause-to-effect relationships. Third, the specialized ICUs were excluded because of the differences in organization, types of procedures, and work pressure. Fourth, the specialized hospitals were excluded because, in China, such hospitals are private hospitals, and their work conditions are very different from those of public hospitals. Finally, the knowledge that this was a survey about burnout might influence the results. This study did not use blinding, but when we designed the questionnaire, we also thought that this might affect the results of the experiment. Therefore, the questionnaire included the question: “Do you know burnout?” As a result, only 206 respondents said they knew about burnout, and 1607 respondents did not know about burnout. In addition, the positive rate of burnout among the investigators who knew about burnout was 81.1% (167/206), and the positive rate of burnout among those who did not know about burnout was 82.3% (1322/1607) (*P* = 0.213). Therefore, in this study, the impact of those participants who knew that this was a burnout survey was limited.

## Conclusions

In conclusion, the prevalence of burnout and severe burnout in intensivists of mainland China is higher compared with other studies reported in other countries. We hope that this survey will bring more attention to burnout in China. The number of children, income, and difficulties in treatment decisions might be factors that affect the prevalence of burnout. Policies should be implemented to improve the well-being of ICU physicians. They could then be surveyed again to examine the effects of those policies.

## Supplementary Information

**Additional file 1.** The questionnaire.

**Additional file 2.** The hospital and respondents in the study.

## Data Availability

The datasets used and/or analyzed during the current study are available from the corresponding author on reasonable request.

## Abbreviations

DP: Depersonalization
EE: Emotional exhaustion
ICUs: Intensive care units
MBI: Maslach Burnout Inventory
PA: Personal accomplishment
