# Supplementary material for: Prevalence of burnout among intensivists in mainland China: a nationwide cross-sectional survey
Source: Crit Care. 2021 Jan 5;25:8. doi: 10.1186/s13054-020-03439-8 (PMC7786985; doi:10.1186/s13054-020-03439-8)
Supplement: Supplementary file 1 — Additional file 1. The questionnaire. [file 13054_2020_3439_MOESM1_ESM.docx]

Supplementary Table 1. The questionnaire.

| Number | Questions | Options |
| --- | --- | --- |
| 1 | Grade of hospital | A tertiary  B secondary |
| 2 | teaching hospital | A yes  B no |
| 3 | Beds in hospital | A <1000  B 1000-2000  C >2000 |
| 4 | Beds in ICU | A <10  B 10-20  C >20 |
| 5 | Sex | A male  B female |
| 6 | Age | A ≤30  B 31-39  C ≥40 |
| 7 | Educational background | A undergraduate  B postgraduate |
| 8 | Academic title | A resident  B attending physicians  C directors |
| 9 | Length of service in ICU | A 1-5y  B 5-10y  C >10y |
| 10 | Marital status | A married  B single or others |
| 11 | Children | A none  B one  C ≥2 children |
| 12 | Satisfaction with income | A yes  B no |
| 13 | Factors affecting income | A Clinical workload  B teaching and research achievement  C Working qualifications  D medical error  E Drug ratio penalty  F others |
| 14 | Uncomfortable symptoms | A dizziness or headache  B insomnia  C fatigue  D muscle intense  E gastrointestinal discomfort  F others |
| 15 | Family history of mental disease | A yes  B no |
| 16 | Chronic diseases | A hypertension  B chronic sleep disorder  C hepatic adipose infiltration  D lumbar disc protrusion  E others  F none |
| 17 | Satisfaction with ICU | A More love  B Less love  C The same as before |
| 18 | Ways to relieve stress | A Sports  B Leisure and recreation  C drink  D Sleep  E smoking  F others |
| 19 | Reasons for choice of ICU | A Affinity  B Distribution  C Others |
| 20 | Weekly working hours | A ≤40 h  B 41-60 h  C >60 h |
| 21 | Night shift | A ≤4 d  B 5-7 d  C >7 d |
| 22 | Commuting time | A ≤0.5 h  B >0.5 h |
| 23 | Holiday | A No holiday  B Having holiday |
| 24 | Attitude to work on holidays | A Acceptance  B Not acceptance  C Indifferent |
| 25 | Compensation for working on holidays | A No compensation  B Having compensation |
| 26 | Worries related to safety | A rare  B often |
| 27 | Most unwilling to do | A having conversation with patient’s family member  B Medical record writing  C rounds  D Taking patient out for examination  E Non-academic conference  F others |
| 28 | Conflict with colleagues | A rare  B often |
| 29 | Difficulties in treatment decisions | A rare  B often |
| 30 | Medical complain affairs | A no  B yes |
| 31 | Most dissatisfaction | A Income  B leadership and work team  C Lack of medical resources  D Working environment  E others |
| 32 | Source of pressures | AClinical practice  B Research and promotion requirements  C Family burden  D personal problem (marriage/fertility)  E social evaluation  F others |
| 33 | Turnover consideration | A no  B yes |
| 34 | Reason for stay | A A love of this work  B No other choice  C Other reasons |
| 35 | Do you know bunrout？ | A yes  B no |
|  | MBI (22questions) | Options |
| 1 | I feel emotionally drained from my work | A never  B some times in a year or less  C once in a month or less  D some times in a month  E once a week  F almost everyday  G everyday |
| 2 | I feel used up at the end of the workday | A never  B some times in a year or less  C once in a month or less  D some times in a month  E once a week  F almost everyday  G everyday |
| 3 | I feel fatigued when i get up in the morning and have to face another day on the job | A never  B some times in a year or less  C once in a month or less  D some times in a month  E once a week  F almost everyday  G everyday |
| 4 | I can easily understand how my recipients feel about things | A never  B some times in a year or less  C once in a month or less  D some times in a month  E once a week  F almost everyday  G everyday |
| 5 | I feel i treat some recipients as if they were impersonal objects | A never  B some times in a year or less  C once in a month or less  D some times in a month  E once a week  F almost everyday  G everyday |
| 6 | Working with people all day is really a strain for me | A never  B some times in a year or less  C once in a month or less  D some times in a month  E once a week  F almost everyday  G everyday |
| 7 | I deal very effectively with the problems of my recipients | A never  B some times in a year or less  C once in a month or less  D some times in a month  E once a week  F almost everyday  G everyday |
| 8 | I feel burned out from my work | A never  B some times in a year or less  C once in a month or less  D some times in a month  E once a week  F almost everyday  G everyday |
| 9 | I feel i’m positively influencing other people’s lives through my work | A never  B some times in a year or less  C once in a month or less  D some times in a month  E once a week  F almost everyday  G everyday |
| 10 | I’ve become more callous toward people since i took this job | A never  B some times in a year or less  C once in a month or less  D some times in a month  E once a week  F almost everyday  G everyday |
| 11 | I worry that this job is hardening me emotionally | A never  B some times in a year or less  C once in a month or less  D some times in a month  E once a week  F almost everyday  G everyday |
| 12 | I feel very energetic | A never  B some times in a year or less  C once in a month or less  D some times in a month  E once a week  F almost everyday  G everyday |
| 13 | I feel frustrated by my job | A never  B some times in a year or less  C once in a month or less  D some times in a month  E once a week  F almost everyday  G everyday |
| 14 | I feel i’m working too hard on my job | A never  B some times in a year or less  C once in a month or less  D some times in a month  E once a week  F almost everyday  G everyday |
| 15 | I don’t really care what happens to some recipients | A never  B some times in a year or less  C once in a month or less  D some times in a month  E once a week  F almost everyday  G everyday |
| 16 | Working with people directly puts too much stress on me | A never  B some times in a year or less  C once in a month or less  D some times in a month  E once a week  F almost everyday  G everyday |
| 17 | I can easily create a relaxed atmosphere with my recipients | A never  B some times in a year or less  C once in a month or less  D some times in a month  E once a week  F almost everyday  G everyday |
| 18 | I feel exhilarated after working closely with my recipients | A never  B some times in a year or less  C once in a month or less  D some times in a month  E once a week  F almost everyday  G everyday |
| 19 | I have accomplished many worthwhile things in this job | A never  B some times in a year or less  C once in a month or less  D some times in a month  E once a week  F almost everyday  G everyday |
| 20 | I feel like i’m at the end of my rope | A never  B some times in a year or less  C once in a month or less  D some times in a month  E once a week  F almost everyday  G everyday |
| 21 | In my work, i deal with emotional problems very calmly | A never  B some times in a year or less  C once in a month or less  D some times in a month  E once a week  F almost everyday  G everyday |
| 22 | I feel recipients blame me for some of their problems | A never  B some times in a year or less  C once in a month or less  D some times in a month  E once a week  F almost everyday  G everyday |

Ee question numbers: 2, 3, 6, 8, 13, 14, 16, 20, 22 (9 questions)

Dp question numbers: 1, 5, 10, 11, 15 (5 questions)

Pa question numbers: 4, 7, 9, 12, 17, 1, 19, 21 (8 questions)
