# Supplementary material for: Prevalence of burnout among intensivists in mainland China: a nationwide cross-sectional survey
Source: Crit Care. 2021 Jan 5;25:8. doi: 10.1186/s13054-020-03439-8 (PMC7786985; doi:10.1186/s13054-020-03439-8)
Supplement: Supplementary file 2 — Additional file 2. The hospital and respondents in the study. [file 13054_2020_3439_MOESM2_ESM.docx]

Supplementary Table 2. The hospital and respondents in the study.

| Regions | Province, municipality, or autonomous region | Hospitals,  n (%) | Teaching hospital affiliated to medical college, n | Hospital level, n  (tertiary/secondary) | Number of beds, n | | Respondents,  n (%) |
| --- | --- | --- | --- | --- | --- | --- | --- |
|  |  |  |  |  | Hospital  (<1000/1000-2000/>2000) | ICU  (<10/10-20/>20) |  |
| North | Heilongjiang | 7 (3.1%) | 5 | 7/0 | 3/3/1 | 3/2/2 | 57 (3.1%) |
|  | Neimenggu | 20 (9.0%) | 6 | 12/8 | 8/10/2 | 7/11/2 | 172 (9.5%) |
|  | Liaoning | 9 (4.0%) | 6 | 9/0 | 0/8/1 | 0/8/1 | 67 (3.7%) |
|  | Jilin | 2 (0.8%) | 2 | 2/0 | 0/1/1 | 0/1/1 | 4 (0.2%) |
|  | Beijing | 4 (1.8%) | 4 | 4/0 | 0/0/4 | 0/0/4 | 31 (1.7%) |
|  | Hebei | 3 (1.3%) | 3 | 3/0 | 0/1/2 | 0/1/2 | 17 (0.9%) |
|  | Shanxi | 15 (6.7%) | 5 | 10/5 | 5/9/1 | 4/10/1 | 121 (6.7%) |
|  | Tianjin | 2 (0.9%) | 2 | 2/0 | 0/1/1 | 0/1/1 | 3 (0.2%) |
|  | **Sum of north** | 62 (27.8%) | 33 | 49/13 | 16/33/13 | 14/34/14 | 472 (26.0%) |
| Northwest | Gansu | 10 (4.5%) | 4 | 7/3 | 4/5/1 | 6/3/1 | 61 (3.4%) |
|  | Ningxia | 13 (5.8%) | 3 | 6/7 | 7/5/1 | 9/3/1 | 73 (4.0%) |
|  | Shanxi | 5 (2.2%) | 2 | 5/0 | 1/3/1 | 0/2/3 | 20 (1.1%) |
|  | Xinjiang | 6 (2.7%) | 4 | 6/0 | 3/2/1 | 1/2/3 | 20 (1.1%) |
|  | Qinghai | 7 (3.1%) | 2 | 3/4 | 4/2/1 | 4/2/1 | 27 (1.5%) |
|  | **Sum of northwest** | 41 (18.4%) | 15 | 27/14 | 19/17/5 | 20/12/9 | 201 (11.1%) |
| Middle of south | Anhui | 7 (3.1%) | 2 | 2/5 | 5/2/0 | 3/3/1 | 74 (4.1%) |
|  | Fujian | 8 (3.6%) | 3 | 3/5 | 5/3/0 | 3/4/1 | 87 (4.8%) |
|  | Henan | 5 (2.2%) | 3 | 3/2 | 2/2/1 | 1/1/3 | 43 (2.4%) |
|  | Hubei | 15 (6.7%) | 10 | 9/6 | 6/5/4 | 2/6/7 | 169 (9.3%) |
|  | Hunan | 10 (4.5%) | 7 | 7/3 | 3/5/2 | 2/1/7 | 91 (5.0%) |
|  | Jiangsu | 6 (2.7%) | 6 | 6/0 | 0/4/2 | 0/2/4 | 59 (3.3%) |
|  | Jiangxi | 9 (1.0%) | 4 | 4/5 | 5/3/1 | 1/3/5 | 107 (5.9%) |
|  | Shandong | 4 (1.8%) | 4 | 4/0 | 0/2/2 | 0/0/4 | 31 (1.7%) |
|  | Zhejiang | 3 (1.3%) | 3 | 3/0 | 0/0/3 | 0/0/3 | 16 (0.9%) |
|  | Shanghai | 3 (1.3%) | 3 | 3/0 | 0/0/3 | 0/0/3 | 10 (0.6%) |
|  | **Sum of middle of south** | 70 (31.4%) | 45 | 44/26 | 26/26/18 | 12/20/38 | 687 (37.9%) |
| Southwest | Guangdong | 8 (3.6%) | 8 | 8/0 | 0/6/2 | 0/6/2 | 62 (3.4%) |
|  | Guangxi | 4 (1.8%) | 1 | 2/2 | 1/3/0 | 1/3/0 | 19 (1.0%) |
|  | Guizhou | 17 (7.6%) | 2 | 5/12 | 11/5/1 | 8/6/3 | 221 (12.2%) |
|  | Yunnan | 12 (5.4%) | 2 | 3/9 | 9/2/1 | 7/3/2 | 117 (6.5%) |
|  | Sichuan | 4 (1.8%) | 4 | 4/0 | 0/2/2 | 0/2/2 | 22 (1.2%) |
|  | Chongqing | 3 (1.3%) | 2 | 3/0 | 0/2/1 | 0/2/1 | 8 (0.4%) |
|  | Hainan | 2 (0.8%) | 1 | 2/0 | 0/2/0 | 0/2/0 | 3 (0.1%) |
|  | **Sum of southwest** | 50 (22.4%) | 20 | 27/23 | 21/22/7 | 16/24/10 | 452 (24.9%) |
| **Total** | | 223 | 113 | 147/76 | 82/98/43 | 62/90/71 | 1813 |
